# Prediction of PD-L1 and CD68 in Clear Cell Renal Cell Carcinoma with Green Learning

**DOI:** 10.3390/jimaging11060191

**Published:** 2025-06-10

**Authors:** Yixing Wu, Alexander Shieh, Steven Cen, Darryl Hwang, Xiaomeng Lei, S. J. Pawan, Manju Aron, Inderbir Gill, William D. Wallace, C.-C. Jay Kuo, Vinay Duddalwar

**Affiliations:** 1 Media Communications Lab, University of Southern California, Los Angeles, CA 90089, USA; yixingwu@usc.edu (Y.W.); jckuo@usc.edu (C.-C.J.K.); 2 Radiomics Lab, Department of Radiology, University of Southern California, Los Angeles, CA 90033, USA; alexander.shieh@med.usc.edu (A.S.); steven.cen@med.usc.edu (S.C.); darryl.hwang@med.usc.edu (D.H.); xiaomeng.lei@med.usc.edu (X.L.); pawan.jogi@med.usc.edu (S.J.P.); 3 Department of Pathology, University of Southern California, Los Angeles, CA 90033, USA; manjuaro@med.usc.edu (M.A.); william.wallace@med.usc.edu (W.D.W.); 4 Institute of Urology, University of Southern California, Los Angeles, CA 90033, USA; igill@usc.edu; 5 Alfred E Mann Department of Biomedical Engineering, USC Viterbi School of Engineering, Los Angeles, CA 90089, USA; 6 Department of Radiology, Los Angeles General Medical Center, Los Angeles, CA 90033, USA

**Keywords:** radiomics, clear cell renal cell carcinoma, PD-L1, CD68, Green Learning, feature selection, biomarkers

## Abstract

Clear cell renal cell carcinoma (ccRCC) is the most common type of renal cancer. Extensive efforts have been made to utilize radiomics from computed tomography (CT) imaging to predict tumor immune microenvironment (TIME) measurements. This study proposes a Green Learning (GL) framework for approximating tissue-based biomarkers from CT scans, focusing on the PD-L1 expression and CD68 tumor-associated macrophages (TAMs) in ccRCC. Our approach includes radiomic feature extraction, redundancy removal, and supervised feature selection through a discriminant feature test (DFT), a relevant feature test (RFT), and least-squares normal transform (LNT) for robust feature generation. For the PD-L1 expression in 52 ccRCC patients, treated as a regression problem, our GL model achieved a 5-fold cross-validated mean squared error (MSE) of 0.0041 and a Mean Absolute Error (MAE) of 0.0346. For the TAM population (CD68+/PanCK+), analyzed in 78 ccRCC patients as a binary classification task (at a 0.4 threshold), the model reached a 10-fold cross-validated Area Under the Receiver Operating Characteristic (AUROC) of 0.85 (95% CI [0.76, 0.93]) using 10 LNT-derived features, improving upon the previous benchmark of 0.81. This study demonstrates the potential of GL in radiomic analyses, offering a scalable, efficient, and interpretable framework for the non-invasive approximation of key biomarkers.

## 1. Introduction

Renal cell carcinoma (RCC) is the most prevalent type of kidney cancer, and clear cell renal cell carcinoma (ccRCC) is the most common subtype of RCC. Advances in immunotherapy have drastically changed the prognosis of ccRCC. However, not all patients respond to these novel treatments. The heterogeneity of the tumor immune microenvironment (TIME) among patients has been thought to play a significant role in the discrepancies in the immunotherapy response. To understand which patients will benefit from specific immunotherapy, many efforts have been put into TIME biomarkers that predict the response to immunotherapies [1]. One trending method for studying the TIME is the use of quantitative multiplex immunofluorescence (mIF), which allows researchers to simultaneously obtain the cell distributions and spatial patterns of several biomarkers. This technique provides scientists with a way to gain a thorough understanding of the TIME by measuring the interactions between multiple cell types in tumors. It offers excellent insight into predicting the prognosis and response to therapy [2]. However, assessments of the TIME that use mIF require tissue samples to be acquired through surgery or image-guided biopsies. The specimens then need to be delicately processed and scanned. Hence, tissue-based assessments of the TIME face limitations due to their invasive nature and the considerable time, effort, and resources required for the tissue samples. In contrast, imaging biomarkers, such as the radiomic features in this study, offer a non-invasive, dynamic approach. These biomarkers can be extracted effectively from computed tomography (CT) scans to assess the progression of the disease and predict the response to treatments in cancer patients. Correlations between radiomics features and TIME measurements have been investigated for cancer types such as lung and breast cancer [3,4,5]. However, for renal cancer, the performance in predicting the TIME from radiomics still involves large room for improvement [6,7]. This could be due to the fact that previous studies have employed limited data or poor prediction methods.

Over the past decade, with deep learning (DL) developments, data-driven methods such as convolutional neural networks (CNNs) have provided a powerful mechanism for feature extraction and classification. This is achieved through an optimization process that involves backpropagation and the minimization of a loss function. Despite their powerful capabilities, these DL tools have faced a lot of limitations, particularly in medical analyses, due to the opaque nature of their decision-making processes, often described as “black-box” mechanisms. In addition, throughout the training phase, deep learning consumes substantial energy and power because of the extensive computational demands of its large-scale models. Moreover, DL models require extensive datasets for training and the maintenance of a vast array of parameters. The requirement for large datasets to effectively train these models may restrict their applicability in medical contexts. However, in critical decision-making scenarios, such as diagnosing cancer in patients, there is a strong preference for methodologies with transparent, understandable, and low-complexity pipelines.

To address the growing resource requirements of contemporary AI systems, the Green Learning (GL) [8] framework offers a novel strategy. The GL framework has three principal advantages: reduced computational complexity, a robust performance compared to that of other models, and enhanced mathematical and statistical transparency. The models within this framework exhibit lower computational demands during training and inference and require fewer training samples while achieving a similar classification or regression performance relative to that of other models. Moreover, GL requires significantly less data than traditional deep learning approaches, making it an ideal framework for applications in the medical field.

Unlike traditional deep neural networks that rely on computational neurons, Green Learning adopts a structured feed-forward approach that distinctly separates the feature extraction from decision learning. This methodology establishes a clear demarcation between the initial data processing stages and the subsequent analytical phases, enhancing the clarity and efficiency of the learning process. The general GL framework consists of three modules: (1) representation learning; (2) supervised feature learning; and (3) supervised decision learning. These components are structured into a sequential cascade, where each process refines the data and generates intermediate outputs that serve as the inputs for the next stage. This design enables individual module optimization and feed-forward training of the overall GL system. Such an architecture allows for independent optimization of each segment.

A key component within this architecture is the supervised feature learning module, which plays a critical role in selecting and refining features. Green Learning introduces a supervised feature learning module specifically designed to select features through an extensive array of representations inherited from previous models. The well-established feature selection approach, namely the discriminant feature test (DFT) and the relevant feature test (RFT) [9], aims to select discriminative features while the minimizing redundancy and reducing the dimensions. While deep learning techniques provide powerful capabilities by integrating feature learning and model training in an end-to-end fashion, the DFT and the RFT offer an alternative strategy explicitly focused on supervised feature selection. The DFT and the RFT use statistical tests to assess the relevance of the features, providing a transparent mechanism for reducing the dimensionality before downstream modeling. As shown in the original paper [9], both the DFT and the RFT handle high-dimensional feature spaces effectively in multiple datasets while maintaining decision accuracy, even in noisy data scenarios. Furthermore, the DFT and the RFT are computationally efficient, making them well suited to large-scale datasets. Further elaboration on this process can be found in Section 2.4 and Section 2.5.

The feature selection component critically evaluates and chooses the most discriminative features from the existing set. Consequently, the effectiveness of the model is heavily dependent on the quality of these selected features. The model’s performance may be compromised if the available features do not represent the overall structure of the data distribution. A novel approach recently introduced by the least-squares normal transform (LNT) [10,11] employs binary projected subspaces of the original label space as estimated target subspaces. This innovative strategy is designed to improve the combination of characteristics, as detailed in Section 2.6.

The Green Learning framework can be applied effectively across a range of machine learning endeavors, including tasks such as image classification [12,13], image deepfake detection [14,15], and prostate region segmentation [16,17]. In our study, we explore GL methodologies for enhancing the analysis of radiomic features and improving the accuracy and reliability of biomarker predictions. This approach takes advantage of the strengths of GL to refine the precision of predictive models in a clinical setting.

In this study, we proposed a GL approach to approximating tissue-based biomarkers using radiomics derived from CT scans. We selected two biomarkers grounded in clinical research: PD-L1 expression and CD68 tumor-associated macrophages (TAMs). They are crucial in the tumor immune microenvironment of ccRCC. PD-L1 is an essential immune checkpoint protein for suppressing the immune system, particularly during cancer progression. One study analyzed 1476 patients with RCC and found that PD-L1 expression was a strong predictor of poor survival [18]. For metastatic ccRCC, another study found that PD-1 and PD-L1 were associated with worse disease characteristics, and the expression of PD-1 was associated with a worse overall survival [19]. CD68 TAMs are a crucial component of the ccRCC TIME. They play multiple roles in the ccRCC TIME, interacting with the tumor and infiltrating T cells. Most importantly, they have been associated with angiogenesis, immunosuppression, and a poor prognosis in ccRCC [20,21,22,23,24]. Therefore, the tumor–TAM interaction can potentially become a biomarker predictive of the response to therapy and the overall prognosis.

To illustrate GL’s versatility in handling classification and regression tasks, we treated the PD-L1 expression level as a regression issue. For CD68+, classification was performed by setting thresholds using the CD68+/PanCK+ ratio to differentiate tumor samples into positive or negative categories.

## 2. Materials and Methods

**Figure 1:**
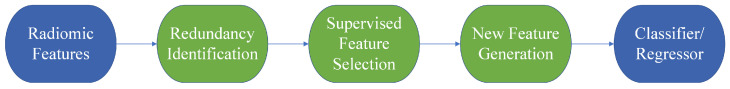
The workflow overview. The pipeline streamlines the radiomic feature processing through redundancy identification, employs the DFT and the RFT for feature selection, and introduces LNT for new feature generation, enhancing the predictive analysis.

As illustrated in Figure 1, our research adopts a structured and modular methodology, incorporating several critical components, which are radiomic feature extraction, feature redundancy identification, supervised feature selection, new feature generation, and utilizing XGBoost (version 2.0.3) [25] for predictions.

### 2.1. The Dataset

Based on patient cohorts and data sources detailed in previous research [7,26,27], this study applies the GL framework to two distinct predictive tasks. For the PD-L1 expression regression task, data from 52 patients were included, and a 5-fold cross-validation strategy (CV) was adopted. For the CD68+ classification task, data from 78 patients were used. The samples were classified according to a CD68 +/PanCK + ratio threshold of 0.4 (ratio > 0.4 as positive, ≤0.4 as negative), a cut-off established in previous work [26]. This resulted in 22 positive and 56 negative samples. A 10-fold CV strategy was used for this task. The CV folds were stratified by class labels to ensure a proportional representation of positive and negative samples in each fold.

A nested cross-validation approach was implemented for both tasks to ensure robust model development and an unbiased performance evaluation. Specifically, within each fold of the outer CV (either 5-fold for regression or 10-fold for classification), the data designated for training were further divided into an internal training set at 80% and an internal validation set at 20%. All of the data-driven model development processes were conducted exclusively using these internal training and validation sets, including redundant feature identification, the DFT/RFT, LNT, and XGBoost hyperparameter optimization. The test set was strictly held out and was not used during any model training or parameter tuning phase.

The final predictive model for each outer fold was then applied to its corresponding held-out test set for inference. The overall reported performance metrics (e.g., the mean squared error, Area Under the Curve) were calculated by averaging the results obtained from all test folds of the outer cross-validation.

### 2.2. Multiplex Immunofluorescence Image Processing

Each stained mIF image was initially captured using PhenoImager (Akoya Biosciences, Inc. (Marlborough, MA, USA)). An expert pathologist identified regions of interest (ROIs) containing viable tumor epithelium using PhenoChart (Akoya Biosciences, Inc.). Then, automatic single-cell phenotype identification and tissue-type segmentation of these ROIs were completed using inForm (Akoya Biosciences, Inc.). Subsequently, the single-cell phenotype counts and tissue area metrics were consolidated at the slice level.

For the PD-L1 regression problem, the expression targets for PD-L1 were approximated as the number of cells positive for PD-L1 divided by the number of cells positive for PanCK. For the CD68 + classification problem, the CD68+/PanCK+ ratio for each specimen was determined by dividing the total count of CD68+ cells by the total count of PanCK+ cells within the segmented tumor epithelium in all ROIs annotated from the specimen. A threshold of CD68+/PanCK+ > 0.4 was used to categorize the samples as positive, while those below this threshold were classified as negative. The previous work [26] searched for all possible cut-off points, and the threshold of 0.4 achieved the highest Area Under the ROC Curve (AUROC) of 0.81. To ensure consistency and allow for a fair comparison, we adopted the same threshold in our Green Learning model to highlight its improved predictive performance.

### 2.3. Multiphase CT Image Processing

Tumor imaging was performed using multiphase contrast-enhanced CT, employing a well-established four-phase imaging protocol [28]. A texture analysis was performed on the CT images utilizing a specialized radiomics pipeline implemented in MATLAB (R2017b). From the segmented tumor volume, 1708 radiomic features were extracted using 13 different texture methods, as described in [29]. They included an intensity/histogram analysis (3D), a Gray Level Size Zone Matrix (GLSZM; 2D and 3D), Laws’ Texture Energy (LTE; 2D and 3D), a Gray Level Run Length Matrix (GLRLM; 2D and 3D), Fast Fourier Transform (FFT; 2D), a Gray Level Dependence Matrix (GLDM; 2D and 3D), Discrete Cosine Transform (DCT; 2D), and a Gray Level Co-Occurrence Matrix (GLCM; 2D and 3D). We see that a large set of features is adopted, highlighting the extensive capability of the pipeline to capture the complexity of tumor textures.

### 2.4. Redundancy Identification

**Figure 2:**
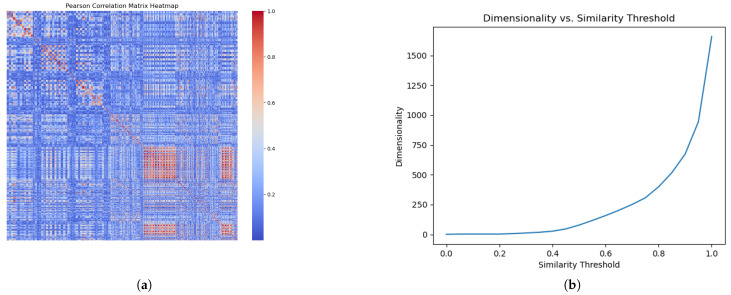
(**a**) Pearson’s correlation matrix heat map; (**b**) the effect of the similarity threshold on the retained feature dimensionality. (**a**) Pearson’s correlation matrix heat map illustrating the degree of correlation between features. Color intensity varies from blue (a correlation coefficient of 0, indicating no correlation) to red (a correlation coefficient of 1, indicating a perfect correlation). (**b**) The impact of varying similarity thresholds on the dimensionality of the remaining features. This graph illustrates how different thresholds influence the number of features retained after redundancy removal. Features are considered similar based on their Pearson’s correlation, and for each pair of similar features, the one with the lower RFT (relevant feature test) loss is retained.

To eliminate redundancy and refine our feature space, we generated a Pearson’s correlation matrix for all input features in the internal training set to identify and remove redundant, highly correlated features. The matrix quantifies the correlation between each feature pair, with the values ranging from −1 to 1. Feature pairs exhibiting an absolute correlation coefficient that exceeded a threshold of 0.9 were identified as redundant. Figure 2a shows a heat map of the correlation matrix. Here, the absolute correlation between any two features, $F_{i}$ and $F_{j}$, is represented as $|r_{ij}|$ within the correlation matrix.

### 2.5. Feature Reduction

Following the evaluation of the feature correlations, our next step is to decrease the dimensionality of the features while preserving essential information. This involves applying the DFT for classification tasks and the RFT for regression tasks to classify features based on their discriminative power. These tests are conducted independently on each outer fold’s internal training and validation sets. The resulting feature rankings and associated loss values guide the subsequent feature selection decisions in this section.

The DFT and the RFT operate by dividing the dynamic range of each one-dimensional (1D) feature into two segments at a specific partition point. They then employ training samples to compute a cost function, for which we use cross-entropy for the DFT and root-mean-squared error (RMSE) for the RFT. Consequently, each feature is assigned a loss value, and these values are ranked in ascending order. In particular, a feature’s discriminative power is inversely reflected by its loss value; for example, features that achieve lower RFT loss values are considered more discriminative.

Once the DFT/RFT loss values were computed for all features on the internal training set, we addressed redundant features. Within each highly correlated feature pair identified in the previous section, we preserved those features with lower DFT/RFT loss values and discarded those with higher ones. Figure 2b illustrates the feature dimension after redundancy removal at various threshold levels. We selected an absolute correlation threshold of 0.9 for the subsequent analysis to optimize the balance between preserving valuable information and eliminating redundancy.

**Figure 3:**
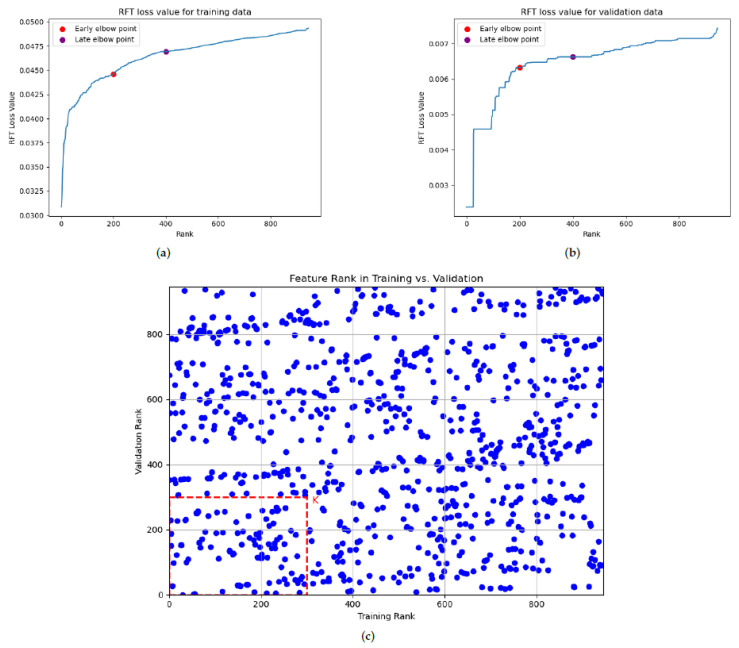
(**a**) The RFT curve of training data for the PD-L1 regression problem, sorted by increasing RFT loss values. Lower RFT loss values indicate the higher discriminative power of the features. (**b**) The RFT curve of validation data for the PD-L1 regression problem, sorted by increasing RFT loss values. Lower RFT loss values indicate the higher discriminative power of the features. (**c**) The RFT ranks of features on the internal training set versus the internal validation set. Features near the origin (0, 0) exhibit robust discriminative power due to high rankings on both sets. The highlighted red square (with the side length *K*) delineates the selected intersection features—those ranking within the top-*K* on both axes.

After eliminating redundant features, the subsequent stage aims to further reduce the dimensionality by identifying the most discriminative subspace of features. This selection is guided by the DFT/RFT loss values of the remaining non-redundant features, which are ranked from smallest (the most discriminative) to largest. Figure 3a,b exemplify these sorted loss value curves, where the x-axis represents the feature rank and the y-axis the corresponding loss value. As highlighted in earlier research [9], identifying early and late elbow points has traditionally guided the determination of the selected feature dimension. For example, in Figure 3a,b, clear early and late elbow points were identified through visual inspection around the top 200 and top 400 ranked features. Features ranked favorably above these points are typically considered strong candidates for the following analysis. We define *K* as a hyperparameter, representing the number of top features to consider. The candidate values for *K* are selected from the range delimited by early (top 200 features) and late (top 400 features) elbow points.

To adopt a more robust selection approach that accounts for potential variance in the data distributions between the training and evaluation phases, independent DFT/RFT processes are performed on the internal training and the corresponding internal validation sets. We aim to identify “intersection features” that consistently exhibit low DFT/RFT loss values (and therefore high ranks) across both datasets. This consistency is a key indicator of a feature’s robust discriminative ability, suggesting that it is less likely to be an artifact of overfitting to the internal training data.

The process of selecting these robust intersection features is visualized in Figure 3c. Each feature is positioned in this plot based on its position in the internal training (x-axis) and internal validation (y-axis). Lower rank values on both axes, thereby placing features closer to the origin, signify a superior discriminative power consistently demonstrated across both datasets, indicating feature robustness. To derive the final feature set, an intersection set was formed for a chosen number of top features *K*. This set comprised features ranked within the top-*K* in both the internal training set’s DFT/RFT results and the internal validation set’s DFT/RFT results. The final operational feature set was subsequently determined by the parameter *K*.

### 2.6. Least-Squares Normal Transform

The least-squares normal transform (LNT), as introduced in [10], represents an innovative approach within Green Learning to generating new discriminant features through linear combinations of existing input features. The derived and original input features are complementary and raw, respectively. These complementary and raw features enhance the model’s discriminative capability. Although the LNT was originally developed for classification tasks, this work adapts its methodology to regression applications, broadening its utility.

To this end, we formulate a least-squares regression problem from an *N*-dimensional (*N*-D) feature space to a one-dimensional (1-D) output space. This involves solving the least-squares normal equation to obtain a normal vector *N*-D, denoted as *A*, the weight vector in our LNT model. For our target vector *T*, which spans *L* training samples, we adapt our approach based on the problem at hand. For the PD-L1 regression issue, *T* consists of PD-L1 expression scalar values. For the CD68+ classification task, *T* embodies binary labels derived from the CD68+/PanCK+ ratio:

(1) AX+B=T,

where $A\in R^{1\times N}$ is the weight vector, $X\in R^{N\times L}$ is the feature matrix, $B\in R^{1\times L}$ is the bias matrix, and $T\in R^{1\times L}$ is the target vector. Since the bias term *B* only introduces a constant shift into the linear transformation, it does not affect the discriminant power of complementary features derived from weighting *A*. Hence, our main focus is the weight vector *A*, derived through the resolution of the normal equation

(2) $$A=TX^{T}{\left(XX^{T}\right)}^{-1}.$$

Once the weight vector *A* is obtained, we can calculate the complementary feature vectors for the training and testing samples. This is achieved by applying the raw feature matrix *x* as the input, following the equation

(3) Ax=p,

where $p\in R^{1\times L}$ is the complementary feature vector.

The equations illustrate that different combinations of raw features lead to different weight matrices *A*, producing different sets of complementary features. Hence, selecting raw features is crucial to enhancing the discriminative power of complementary features. Features exhibiting low loss values in the DFT/RFT are more likely to yield discriminative complementary features. Consequently, the robust intersection features, those top*K* features identified in the previous section as consistently high-ranking across internal training and validation sets, are selected as the input for the LNT model.

To effectively utilize these raw features, a segmented rank-based grouping strategy organizes them into several diverse subgroups. These input features, already classified by their DFT/RFT loss values, are assigned into subgroups based on a predefined stride, denoted as *s*. Specifically, for each initial rank offset *i* (from 1 to *s*), the *i*-th subgroup is assembled by selecting the features at ranks i,i+s,i+2s,…,i+ks (where *k* = 0, 1, 2,…). Each subgroup is then processed using the LNT method to produce a complementary feature. This approach facilitates the generation of multiple novel features, thereby enriching the feature dimension, and maximizes the information integration by combining features of varied original ranks within each subgroup.

### 2.7. Decision Learning

After completing the feature dimension reduction and generating new complementary features, we concatenated the selected DFT/RFT features with the newly generated complementary features. We used XGBoost for decision learning, addressing regression and classification challenges. For the PD-L1 regression, our model was validated through 5-fold cross-validation, assessing the performance through the MSE, RMSE, and MAE. For the CD68+/PanCk+ classification, we applied a 10-fold cross-validation approach, aligning with the benchmarks set in the prior study [26]. We evaluated the model’s performance by measuring the Receiver Operating Characteristic (ROC) curve and the Area Under the ROC Curve (AUC) alongside 95% confidence intervals, ensuring a comprehensive evaluation of its predictive capability and consistency. The reduction in the feature dimension and the generation of new complementary features were repeated for each training sample without testing data during cross-validation to prevent information leakage. After hyperparameter fine-tuning, the optimal results are achieved when the maximum tree depth is limited to 3, and the number of trees is below 500. This outcome demonstrates that our model can achieve an excellent performance while ensuring simplicity.

## 3. Results

For the PDL1 regression task, Table 1 compares the optimal average MSE, RMSE, and MAE with their standard deviations, obtained using 5-fold cross-validation. This comparison includes our GL model under different conditions and three benchmarking models: (1) the Random Forest (100 estimators), (2) AdaBoost (a Decision Tree base estimator with a max depth of 3, 300 estimators), and (3) ElasticNet (α=5; $l_{1}$ ratio = 0.2; max_iter = 5000). It also includes an ablation study illustrating the impact of the feature selection and the LNT. The results show how each component contributes to the overall performance improvement. Specifically, the comparison reveals that our model, with feature selection and the LNT applied, consistently outperforms the baseline models in terms of its MSE, RMSE, and MAE, underscoring the effectiveness of our approach.

Initially, without any feature selection or transformation, the model recorded an MSE of 0.00608, an RMSE of 0.0709, and an MAE of 0.0450. However, the introduction of feature selection led to significant improvements, indicating that filtering the features based on their correlation with the target variable effectively reduced noise and enhanced the focus on relevant predictors. Feature selection alone resulted in substantial performance gains, already exceeding the baseline models in terms of the MSE.

Incorporating the LNT alongside feature selection further refines the performance, reducing the MSE to 0.00408, the RMSE to 0.05708, and the MAE to 0.03460. This combination showcases the value of generating discriminative complementary features via the LNT and selecting meaningful raw features through correlation filtering, leading to a more accurate and robust regression model.

In particular, our model, which incorporates feature selection and the LNT, notably demonstrates a strong and consistent predictive performance. Table 1 reports standard deviations that quantify each model’s performance variability in the different data folds. Our model achieves a highly competitive average for the MAE metric. In addition, its standard deviation (±0.00963) is the lowest among all baseline models, reflecting a strong predictive capability. The results underscore the importance of strategic feature selection and transformation in achieving precise and reliable regression outcomes.

For the CD68 classification task, Table 2 presents the AUC scores and their corresponding 95% confidence intervals for the CD68+ classification problem. In this context, the result of the feature reduction stage is the intersection of the top-*K* ranked features obtained from the DFT rankings of both the internal training and the internal validation sets, and *N* specifies the number of new complementary features derived from the LNT. Because our method leverages intersection features identified from the training and validation data, the input feature dimension for XGBoost is adaptive across different folds. Remarkably, we observed that the highest AUC score, 0.85, is achieved using only 10 LNT-derived features without the original feature input. This setting also results in the smallest 95% confidence interval, which highlights the precision and robustness of the method. After the redundancy identification and removal step in the LNT process, these 10 LNT features were generated by entering the remaining features. The number of these input features for the LNT varied from approximately 1230 to 1250 across the 10 different cross-validation folds.

**Figure 4:**
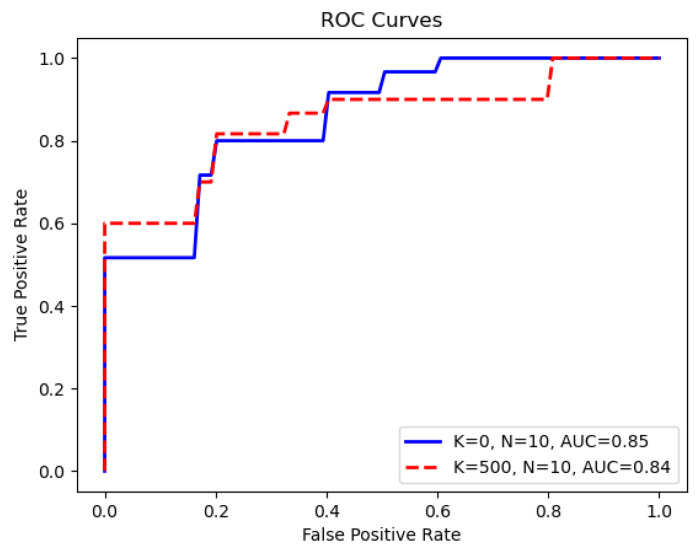
Average ROC curves from 10-fold cross-validation for *K* = 500 and *K* = 0, with *N* = 10 complementary features.

Comparing our current results with those of previous work on the same dataset (summarized in Table 3, sourced from [26]), our Green Learning (GL) model demonstrates a notable improvement, achieving an AUC of 0.85, which surpasses the previous benchmark of 0.81. In particular, the utilization of just 10 complementary features simplified the model and ensured a narrower confidence interval, affirming the robustness of the Green Learning model in the 10-fold cross-validation process. Figure 4 illustrates the average ROC curves for the scenarios where K=500 and K=0.

## 4. Discussion

The redundancy identification and removal process evaluates the linear relationship between each pair of features. This crucial step uncovers pairs of highly correlated features, pinpointing redundancies that do not contribute new information to our dataset. Figure 2a displays the complete correlation matrix as a heat map. Within it, sections highlighted in orange indicate feature pairs with a high correlation. Identifying such pairs is crucial to reducing the feature space without sacrificing significant informational content, while streamlining the model by reducing its complexity.

In Figure 2b, the horizontal axis represents the absolute correlation threshold for identifying highly correlated feature pairs, and the vertical axis indicates the remaining dimensions of the feature. Setting the threshold to 1 implies that no feature pairs are considered highly correlated, resulting in no feature exclusion; conversely, a threshold of 0 means that all pairs are viewed as highly correlated. Notably, adjusting the threshold to 0.95 yields a substantial decrease in the feature dimensions from 1708 to 945, and lowering it further to 0.90 results in a reduction to 674 dimensions, highlighting the presence of significant correlations within the feature set. This visualization demonstrates the effective refinement of our feature set, achieving a more streamlined and potent dataset by applying a threshold strategy.

**Figure 5:**
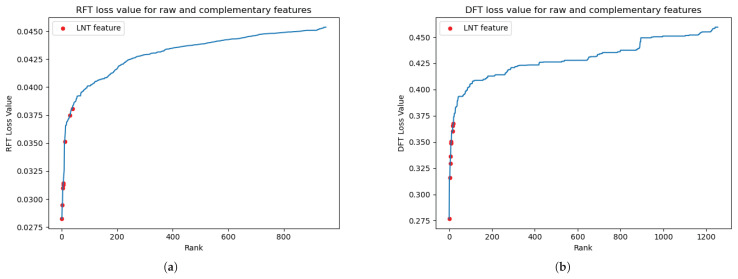
(**a**) The feature importance plot of the PD-L1 regression experiment: the RFT ranks of raw and 8 LNT complementary features. The red points highlight the ranks of the LNT features, which are positioned before the elbow point, indicating their low RFT loss values. This demonstrates that all of the newly generated features are highly discriminative. (**b**) The feature importance plot of the CD68 classification experiment: the DFT ranks of raw and 10 LNT complementary features with a stride *s* = 5. The red points highlight the ranks of the LNT features, which are positioned before the elbow point, indicating their low DFT loss values. This demonstrates that all of the newly generated features are highly discriminative.

We have shown the discriminative power of complementary features derived from the LNT in previous work [27]. Unlike earlier studies in which only a single LNT feature was incorporated, this study introduced multiple LNT-generated complementary features, significantly enhancing the model’s prediction ability. Illustrated in Figure 5a, these newly introduced LNT features notably reduce the minimum value of the RFT loss, compared to the minimum value of the raw features depicted in Figure 3a. Specifically, the lowest loss value for the LNT features is 0.025, in contrast to 0.03 in the raw feature space. Remarkably, the eight newly introduced features fall before the critical elbow point, underscoring the LNT model’s enhanced discriminative capability in selectively chosen raw features. This enhancement is also consistently observed in the CD68 classification scenario. As Figure 5b shows, the ten complementary features significantly exceed the early elbow point in the ranking, reaffirming the robustness of the LNT model and the improved discriminative capacity by introducing multiple complementary features.

**Figure 6:**
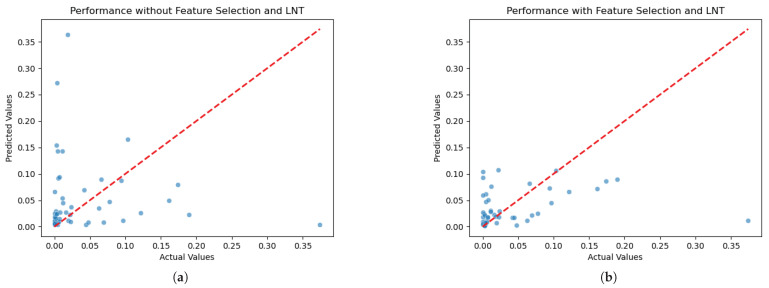
Comparison of PD-L1 regression performance with and without supervised feature selection and LNT. (**a**) Scatter plot of actual versus predicted PD-L1 expression levels without supervised feature selection and LNT. (**b**) Scatter plot of actual versus predicted PD-L1 expression levels with supervised feature selection and LNT. In both plots, each point represents a predicted value, with actual values on the x-axis and predicted values on the y-axis. The red dashed line indicates the ideal line of perfect prediction.

To visualize the overall data distribution of the PD-L1 regression problem, Figure 6a,b illustrate the distribution of the actual versus predicted PD-L1 expression levels, with and without the incorporation of supervised feature selection and the LNT. These visual comparisons highlight the impact of our methodological improvements on model accuracy. In Figure 6a, the scatter of points indicates greater variance in the prediction accuracy. This suggests that the model without feature selection and the LNT struggles to predict higher expression levels consistently and accurately. In contrast, Figure 6b shows a significant improvement in the precision of the predictions, as evidenced by the clustering of points near the perfect prediction line. Implementing redundancy identification, supervised feature selection, and the LNT notably reduces the mean squared error (MSE), bringing the predictions closer to the actual values.

**Figure 7:**
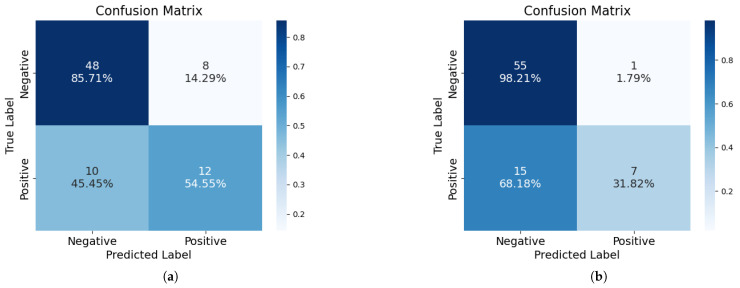
Confusion matrices derived from aggregated predictions on the test data of the 10-fold cross-validation for the CD68 classification task. (**a**) The Green Learning model using 10 LNT features. (**b**) The Random Forest model.

For the CD68 classification problem, our study uses the same dataset as that in the previous work of Shieh et al. [26], characterized by a significant imbalance, with 56 negative and 22 positive samples. Table 3 presents the methods and the corresponding AUC results of this earlier study. In comparison, as shown in Table 2, the AUC values for our models in various settings exceed those reported previously, demonstrating the improved performance of our model in managing this highly unbalanced binary classification challenge. Figure 7a provides a detailed visualization of our Green Learning model’s results, while Figure 7b presents the results of the Random Forest model. A comparison between these two figures highlights the clear advantage of our model in managing imbalanced data, particularly in the minority (positive) samples. Specifically, the GL model achieves a sensitivity of 54.55% (correctly identifying 12 of 22 positive samples) for this minority class. This is substantially higher than the 31.82% sensitivity (7 of 22) achieved by the RF model, favoring the majority class. While the RF model demonstrates a higher specificity for the majority negative class (RF: 98.21% vs. GL: 85.71%), the GL model’s superior ability to detect positive instances points to a more balanced classification performance crucial for imbalanced datasets. This underscores the effectiveness of our approach in robustly predicting the minority class.

However, our research work is limited by the size of the available datasets. It is apparent from Figure 6b that there is still an outlier that the model does not accurately predict. This outlier, a sample with a PD-L1 value greater than 0.2, illustrates a limitation due to the small size of our dataset. With only one sample in this higher range, the model lacks sufficient data to learn from, making accurate predictions challenging for such outlier values. While our comprehensive approach leads to improved overall accuracy and a better fit to the data trends compared to other models, it is recognized in Figure 6b that some individual predictions still exhibit noticeable deviations from their actual values throughout the observed range. This suggests that although the model’s ability to capture the underlying signal is significantly improved, refining the precision for every individual estimate remains an avenue for future investigation. Ultimately, this shows the need for larger datasets in future studies to enhance the robustness of the model across the full spectrum of PD-L1 expression levels. Similarly, in the CD68 classification challenge, the limited size of the dataset constrains the model’s ability to accurately predict unbalanced data during inference. Despite the challenges posed by small and highly imbalanced datasets, which are prevalent in medical applications, the Green Learning framework offers a robust solution. This framework effectively addresses issues related to the limited size and imbalance of the data and surpasses traditional machine learning methods in providing statistical and mathematical transparency. It improves the reliability and interpretability of the model outcomes, which is crucial for medical decision-making.

## 5. Conclusions

In this study, we demonstrated the power of GL in predicting the expression values of ccRCC PD-L1 and the binary classification of CD68 through radiomic features. Our model significantly streamlines the process of determining biomarker expression levels, which is traditionally a labor-intensive task that requires a significant time commitment from experts for medical imaging analyses. Using radiomic characteristics, our approach offers the potential to significantly reduce the need for manual intervention and extensive pathological review, thus enhancing the overall efficiency of clinical imaging analyses. The Green Learning pipeline is modular, transparent, interpretable, and lightweight. Our empirical findings underscore the utility of GL in scenarios characterized by high-dimensional but limited datasets. Initially, the radiomic features extracted from CT images were refined using correlation coefficients and the DFT/RFT to minimize the dimensionality of the features. Subsequently, the least-squares normal transform (LNT) module systematically clustered the raw features into several subgroups and generated new discriminative features for each subgroup. Combining selected raw features and these complementary features was then harnessed to train an XGBoost regressor or classifier. Executed in a feed-forward fashion, this pipeline stands out for its mathematical clarity and modular architecture. However, the limited size of our dataset constitutes a constraint to our study. We aim to validate and possibly enhance this pipeline in larger datasets and diverse diseases, strengthening its applicability and robustness.

## Figures and Tables

**Table 1:** Performance comparison of baseline models and ablation study for PD-L1 Regression.

Regressor	Feature Selection	LNT	MSE	RMSE	MAE
Random Forest			0.00449 ± 0.00438	0.06118 ± 0.02725	0.04277 ± 0.01127
AdaBoost			0.00428 ± 0.00486	0.05761 ± 0.03098	0.03392 ± 0.01489
ElasticNet			0.00456 ± 0.00461	0.06074 ± 0.02943	0.04501 ± 0.01362
XGBoost			0.00608 ± 0.00487	0.07090 ± 0.02795	0.04560 ± 0.01639
XGBoost	√		0.00422 ± 0.00445	0.05760 ± 0.02824	0.03644 ± 0.01191
XGBoost	√	√	0.00408 ± 0.00446	0.05708 ± 0.02861	0.03460 ± 0.00963

Note: Values are shown as the mean ± standard deviation. √ indicates the inclusion of the corresponding component.

**Table 2:** AUC performance with 95% confidence intervals for CD68 level classification using XGBoost across various feature dimensions. *K* denotes the elbow points selected in the training and validation dataset, and *N* denotes the complementary feature dimension.

Top K+N Features	Input Feature Dimension for XGBoost	AUC
300 + 10	115–170	0.83 [0.73,0.92]
400 + 10	180–230	0.82 [0.72,0.92]
500 + 10	200–310	0.84 [0.72,0.92]
0 + 10	10	0.85 [0.76,0.93]

**Table 3:** AUC performance with 95% confidence intervals for CD68 level classification from previous work [26].

Methods from Previous Work	AUC
Random Forest	0.81 [0.69,0.92]
AdaBoost	0.74 [0.6,0.88]
ElasticNet	0.78 [0.64,0.91]

## Data Availability

The data generated or analyzed during this study can be made available by the corresponding author on request, and institutional review deidentified data may be made available per the university’s IRB rules and regulations for private review access.

## Abbreviations

The following abbreviations are used in this manuscript:

ccRCC	Clear Cell Renal Cell Carcinoma
TIME	Tumor Immune Microenvironment
CT	Computed Tomography
GL	Green Learning
MRI	Magnetic Resonance Imaging
DFT	Discriminant Feature Test
RFT	Relevant Feature Test
LNT	Least-Squares Normal Transform
MSE	Mean Squared Error
MAE	Mean Absolute Error
AUROC	Area Under the Receiver Operating Characteristic
RF	Random Forest
SVM	Support Vector Machine
CNN	Convolutional Neural Network
RMSE	Root-Mean-Squared Error

## References

[1] Simonaggio A., Epaillard N., Pobel C., Moreira M., Oudard S., Vano Y.A. (2021). Tumor Microenvironment Features as Predictive Biomarkers of Response to Immune Checkpoint Inhibitors (ICI) in Metastatic Clear Cell Renal Cell Carcinoma (mccRCC). Cancers.

[2] Chevrier S., Levine J.H., Zanotelli V.R.T., Silina K., Schulz D., Bacac M., Ries C.H., Ailles L., Jewett M.A.S., Moch H. (2017). An Immune Atlas of Clear Cell Renal Cell Carcinoma. Cell.

[3] Wang J.H., Wahid K.A., van Dijk L.V., Farahani K., Thompson R.F., Fuller C.D. (2021). Radiomic biomarkers of tumor immune biology and immunotherapy response. Clin. Transl. Radiat. Oncol..

[4] Kang C.Y., Duarte S.E., Kim H.S., Kim E., Park J., Lee A.D., Kim Y., Kim L., Cho S., Oh Y. (2022). Artificial Intelligence-based Radiomics in the Era of Immuno-oncology. Oncologist.

[5] Kothari G. (2022). Role of radiomics in predicting immunotherapy response. J. Med. Imaging Radiat. Oncol..

[6] Varghese B., Cen S., Zahoor H., Siddiqui I., Aron M., Sali A., Rhie S., Lei X., Rivas M., Liu D. (2022). Feasibility of using CT radiomic signatures for predicting CD8-T cell infiltration and PD-L1 expression in renal cell carcinoma. Eur. J. Radiol. Open.

[7] Shieh A.T.W., Cen S.Y., Varghese B., Hwang D.H., Lei X., Gurumurthy K., Siddiqi I., Aron M., Gill I., Wallace W.D. Bridging radiomics to tumor immune microenvironment assessment in clear cell renal cell carcinoma. Proceedings of the 18th International Symposium on Medical Information Processing and Analysis, SPIE.

[8] Kuo C.C.J., Madni A.M. (2022). Green learning: Introduction, examples and outlook. J. Vis. Commun. Image Represent..

[9] Yang Y., Wang W., Fu H., Kuo C.C.J. (2022). On supervised feature selection from high dimensional feature spaces. APSIPA Trans. Signal Inf. Process..

[10] Wang X., Mishra V.K., Kuo C.C.J. Enhancing Edge Intelligence with Highly Discriminant LNT Features. Proceedings of the 2023 IEEE International Conference on Big Data (BigData), IEEE.

[11] Wang X., Wu Y., Li H., Mishra V.K., Kuo C.C.J. A Statistics-based Feature Generation (SFG) Method: Theory and Applications. Proceedings of the 2024 IEEE International Conference on Big Data (BigData), IEEE.

[12] Chen Y., Kuo C.C.J. (2020). Pixelhop: A successive subspace learning (ssl) method for object recognition. J. Vis. Commun. Image Represent..

[13] Chen Y., Rouhsedaghat M., You S., Rao R., Kuo C.C.J. Pixelhop++: A small successive-subspace-learning-based (ssl-based) model for image classification. Proceedings of the 2020 IEEE International Conference on Image Processing (ICIP), IEEE.

[14] Zhu Y., Wang X., Chen H.S., Salloum R., Kuo C.C.J. A-PixelHop: A Green, Robust and Explainable Fake-Image Detector. Proceedings of the ICASSP 2022—2022 IEEE International Conference on Acoustics, Speech and Signal Processing (ICASSP).

[15] Zhu Y., Wang X., Salloum R., Chen H.S., Kuo C.C.J. (2022). RGGID: A Robust and Green GAN-Fake Image Detector. APSIPA Trans. Signal Inf. Process..

[16] Kaneko M., Magoulianitis V., Ramacciotti L.S., Raman A., Paralkar D., Chen A., Chu T.N., Yang Y., Xue J., Yang J. (2024). The Novel Green Learning Artificial Intelligence for Prostate Cancer Imaging: A Balanced Alternative to Deep Learning and Radiomics. Urol. Clin..

[17] Kaneko M., Cacciamani G.E., Yang Y., Magoulianitis V., Xue J., Yang J., Liu J., Lenon M.S.L., Mohamed P., Hwang D.H. (2023). MP09-05 Automated prostate gland and prostate zones segmentation using a novel mri-based machine learning framework and creation of software interface for users annotation. J. Urol..

[18] Möller K., Fraune C., Blessin N.C., Lennartz M., Kluth M., Hube-Magg C., Lindhorst L., Dahlem R., Fisch M., Eichenauer T. (2021). Tumor cell PD-L1 expression is a strong predictor of unfavorable prognosis in immune checkpoint therapy-naive clear cell renal cell cancer. Int. Urol. Nephrol..

[19] Ueda K., Suekane S., Kurose H., Chikui K., Nakiri M., Nishihara K., Matsuo M., Kawahara A., Yano H., Igawa T. (2018). Prognostic value of PD-1 and PD-L1 expression in patients with metastatic clear cell renal cell carcinoma. Urol. Oncol..

[20] Dannenmann S., Thielicke J., Stockli M., Matter C., von Boehmer L., Cecconi V., Hermanns T., Hefermehl L., Schraml P., Moch H. (2013). Tumor-associated macrophages subvert T-cell function and correlate with reduced survival in clear cell renal cell carcinoma. Oncoimmunology.

[21] Wang C., Hong T., Wang Y., Gan S., Wang Q., Li J., Zuo L., Cui X. (2020). Integration of intratumoral RASSF10 expression and tumor-associated macrophages into the established clinical indicators better predicts the prognosis of clear cell renal cell carcinoma patients. Oncoimmunology.

[22] Cros J., Sbidian E., Posseme K., Letierce A., Guettier C., Benoît G., Ferlicot S. (2016). Nestin expression on tumour vessels and tumour-infiltrating macrophages define a poor prognosis subgroup of pt1 clear cell renal cell carcinoma. Virchows Arch..

[23] Chakiryan N.H., Kimmel G.J., Kim Y., Hajiran A., Aydin A.M., Zemp L., Katende E., Nguyen J., Lopez-Blanco N., Chahoud J. (2021). Spatial clustering of CD68+ tumor associated macrophages with tumor cells is associated with worse overall survival in metastatic clear cell renal cell carcinoma. PLoS ONE.

[24] Hajiran A., Chakiryan N., Aydin A., Zemp L., Nguyen J., Laborde J., Chahoud J., Spiess P., Zaman S., Falasiri S. (2021). Reconnaissance of tumor immune microenvironment spatial heterogeneity in metastatic renal cell carcinoma and correlation with immunotherapy response. Clin. Exp. Immunol..

[25] Chen T., Guestrin C. Xgboost: A scalable tree boosting system. Proceedings of the 22nd ACM SIGKDD International Conference on Knowledge Discovery and Data Mining.

[26] Shieh A., Cen S.Y., Varghese B.A., Hwang D., Lei X., Setayesh A., Siddiqi I., Aron M., Dsouza A., Gill I.S. (2024). Radiomics Correlation to CD68+ Tumor-Associated Macrophages in Clear Cell Renal Cell Carcinoma. Oncology.

[27] Wu Y., Shieh A., Cen S., Varghese B., Hwang D.H., Wallace W., Kuo C.C.J., Duddalwar V. Discriminant Radiomic Feature Selection for PD-L1 Prediction in Clear Cell Renal Cell Carcinoma. Proceedings of the 2023 19th International Symposium on Medical Information Processing and Analysis (SIPAIM), IEEE.

[28] Demirjian N.L., Varghese B.A., Cen S.Y., Hwang D.H., Aron M., Siddiqui I., Fields B.K.K., Lei X., Yap F.Y., Rivas M. (2022). CT-based radiomics stratification of tumor grade and TNM stage of clear cell renal cell carcinoma. Eur. Radiol..

[29] Varghese B.A., Hwang D., Cen S.Y., Lei X., Levy J., Desai B., Goodenough D.J., Duddalwar V.A. (2021). Identification of robust and reproducible CT-texture metrics using a customized 3D-printed texture phantom. J. Appl. Clin. Med. Phys..

